# Supplementary material for: Influence of energy gap between charge-transfer and locally excited states on organic long persistence luminescence
Source: Nat Commun. 2020 Jan 10;11:191. doi: 10.1038/s41467-019-14035-y (PMC6954229; doi:10.1038/s41467-019-14035-y)
Supplement: Supplementary file 1 — Supplementary information [file 41467_2019_14035_MOESM1_ESM.pdf]

Supplementary Information

Influence of energy gap between charge-transfer and locally excited states on organic long persistence luminescence

Lin et al.

1. Supplementary Methods

Quantum-chemistry calculations of the dipole moment. All dipole moment values were calculated using the program Gaussian 09. The *cis*- and *trans*-conformations of DMDTB in the ground state were determined using density functional theory (DFT) at the B3LYP/6-31G(d) level. Geometry optimizations of the ground state were performed using DFT at the PBE1PBE/ma-Def2TZVP level. The S_1 and T_1 states, as well as the oxidation and reduction states of the investigated three donors and PPT, were calculated at the same level.

Synthesis of *N,N'*-dimethyl-*N,N'*-ditolylbenzidine (DMDTB).

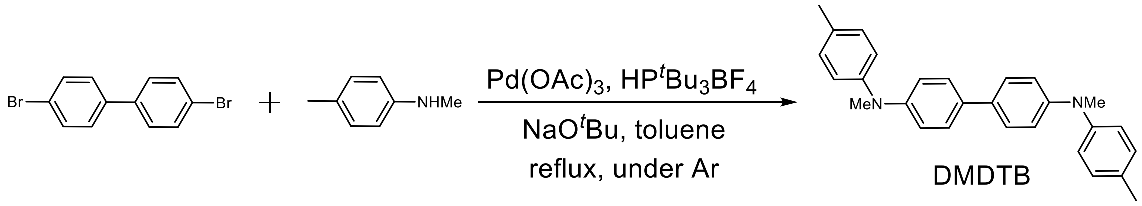

A mixture of *4,4'*-dibromobiphenyl (2.0 g, 6.4 mmol), *N*-methyl-*p*-toluidine (2 mL, 16 mmol), $Pd(OAc)_2$ (28.6 mg, 0.13 mmol), tri-*t*-butylphosphine tetrafluoroborate (42.1 mg, 0.15 mmol) and NaO*t*Bu (2.5 g, 26 mmol) was refluxed overnight in 30 mL dry degassed toluene under argon. After cooling to room temperature, the mixture was poured into water, extracted with CH_2Cl_2, washed with water, and then dried with Na_2SO_4. A pinkish-white product was obtained by column chromatography under dark conditions using $CHCl_3$:hexane = 1:3 as eluent (2.27 g, 90% yield). A white pure product was obtained by purification by train-sublimation. ^{1}H NMR (500 MHz, chloroform-*d*) δ 7.51 – 7.33 (m, 4H), 7.20 – 7.08 (app d, 4H), 7.07 – 7.00 (m, 4H), 7.00 – 6.91 (m, 4H), 3.31 (s, 6H), 2.33 (s, 6H). ^{13}C NMR (126 MHz, $CDCl_3$) δ 147.98, 146.49, 132.47, 132.03, 129.90, 127.01, 122.53, 118.36, 40.36, 20.75. APCI-MS *m/z*: 392.22 $[M]^+$. Element Analysis (calculated & found for $C_{28}H_{28}N_2$): C (85.67%, 85.68%), H (7.19%, 7.21%), N (7.14%, 7.17%).

2. Supplementary Figures and Tables

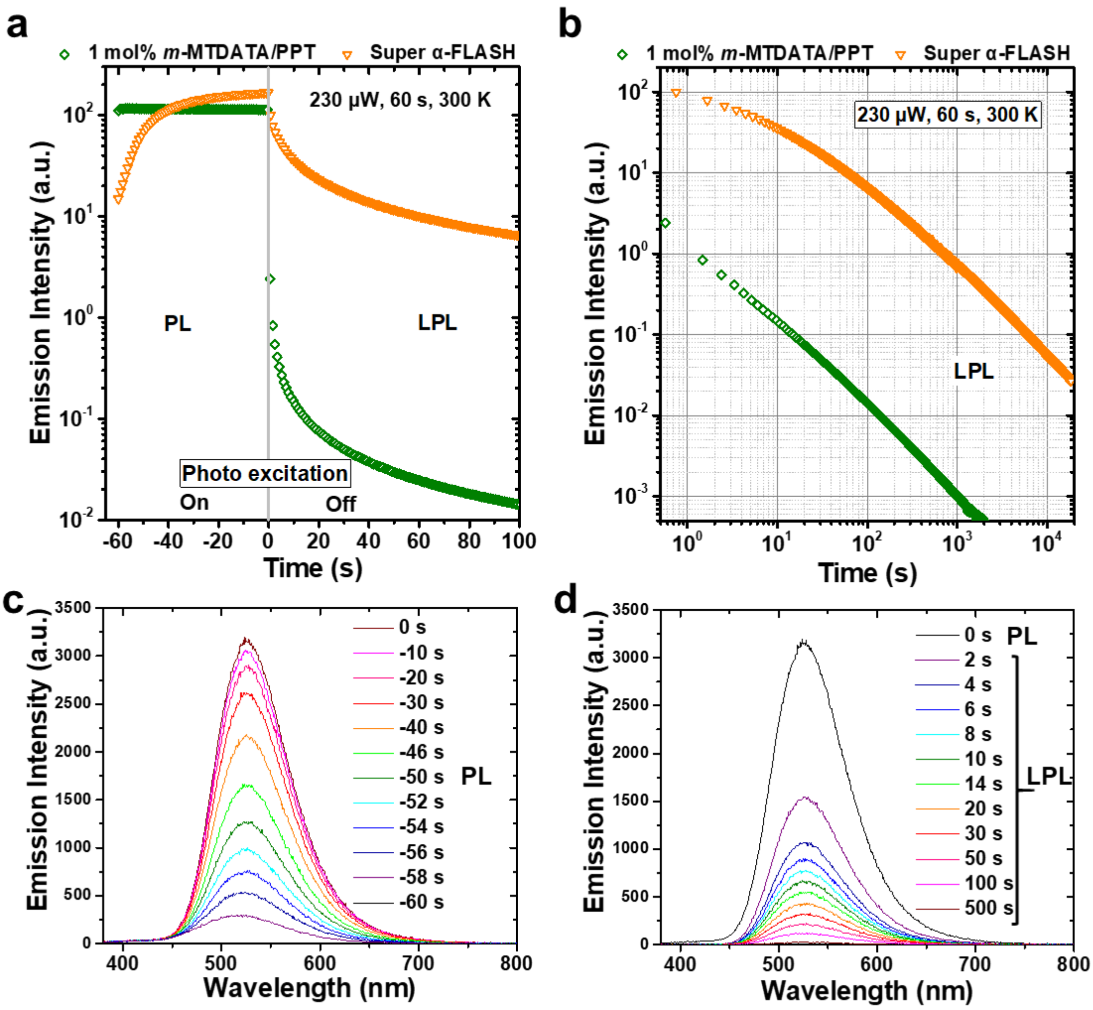

Supplementary Figure 1. Comparison between OLPL and inorganic LPL system. Semi-logarithmic plots (**a**) and logarithmic plots (**b**) of the emission decay profiles of the reported OLPL system (1 mol% *m*-MTDATA/PPT)[1] and a commercial inorganic LPL material (Super α-Flash, LTI corporation, Japan). Emission spectra during photoexcitation (**c**) and after the excitation (**d**) of inorganic LPL material. All samples were 1 cm^2 and were excited for 60 s by a 340-nm LED source with the same power of 230 μW at 300 K.

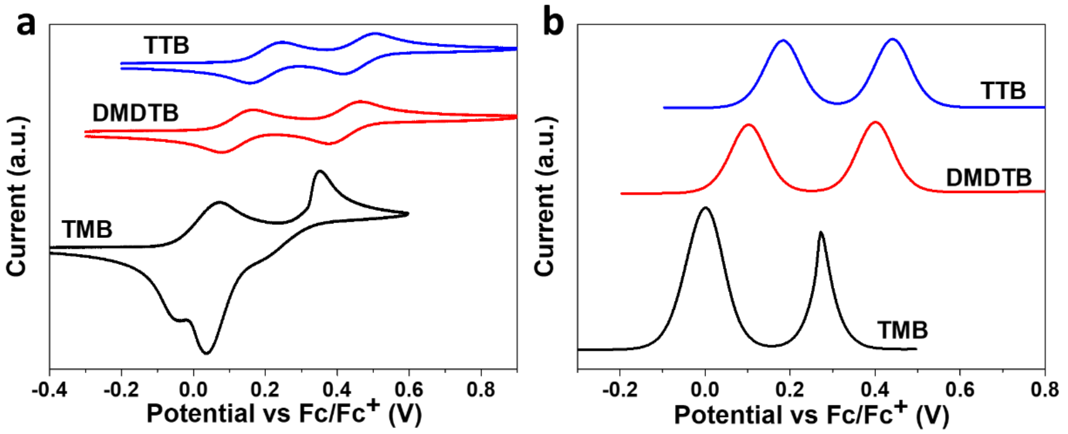

Supplementary Figure 2. Cyclic voltammograms. Cyclic voltammetry (CV) (**a**) and differential pulse voltammetry (DPV) (**b**) curves of TMB, DMDTB, and TTB. The first redox potential of donors (vs. Fc/Fc^+) is 0.00 V (TMB), 0.10 V (DMDTB) and 0.18 V (TTB), respectively.

Supplementary Table 1. The m values for fits of $I(t) \sim t^{-m}$ to the decay curves over different time periods.

Doped films	m (10–30 s)	R^2	m (30–50 s)	R^2	m (50–100 s)	R^2	m (100–300 s)	R^2
TMB/PPT	−0.99	0.9997	−1.08	0.9999	−1.15	0.9999	−1.24	0.9998
DMDTB/PPT	−0.93	0.9998	−1.00	0.9999	−1.05	0.9999	−1.12	0.9996
TTB/PPT	−0.89	0.9997	−0.97	0.9999	−1.04	0.9998	−1.08	0.9993

The m values are from the slopes of double log plots in Figure 1c. "R^2" is the coefficient of determination.

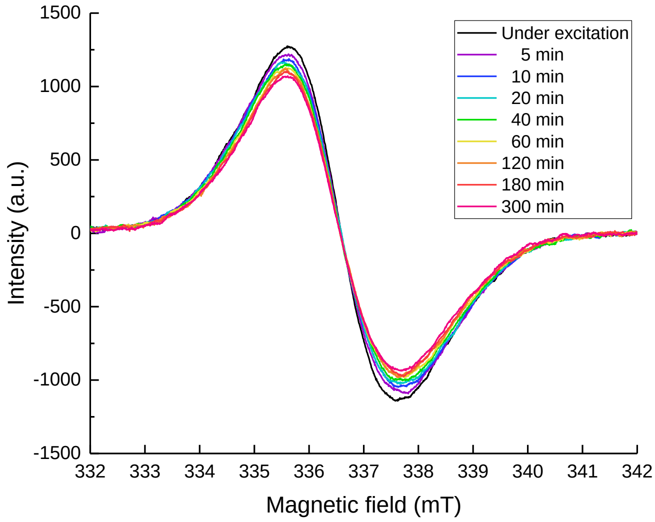

Supplementary Figure 3. Time-resolved electron spin resonance (ESR) signal of the TMB/PPT system. The ESR signal clearly increases after photo-excitation due to the charge separation process. The ESR signal is gradually decreased by time.

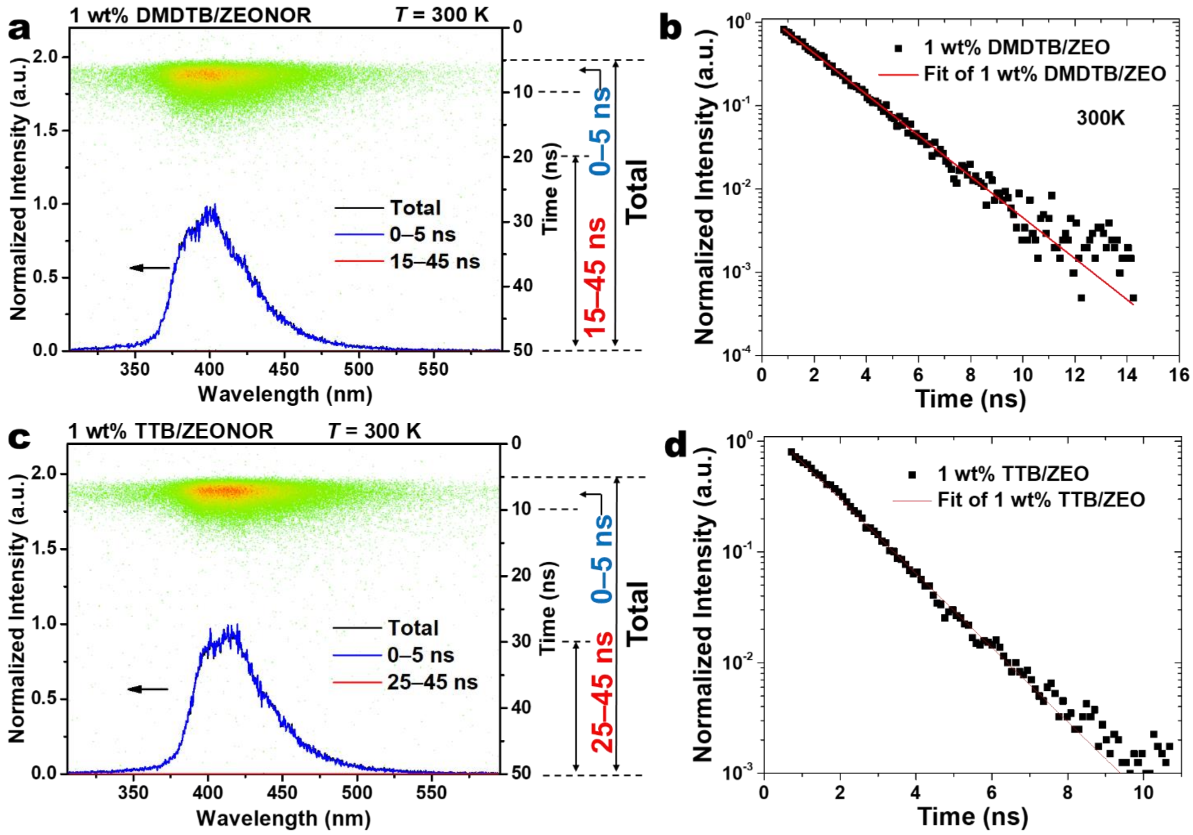

Supplementary Figure 4. Transient emission data of 1 wt% DMDTB/ZEONOR and TTB/ZEONOR films. The streak images, transient emission spectra (**a, c**), and emission decay profiles (**b, d**) of 1 wt% DMDTB/ZEONOR and TTB/ZEONOR films on a 50-microsecond timescale at 300 K.

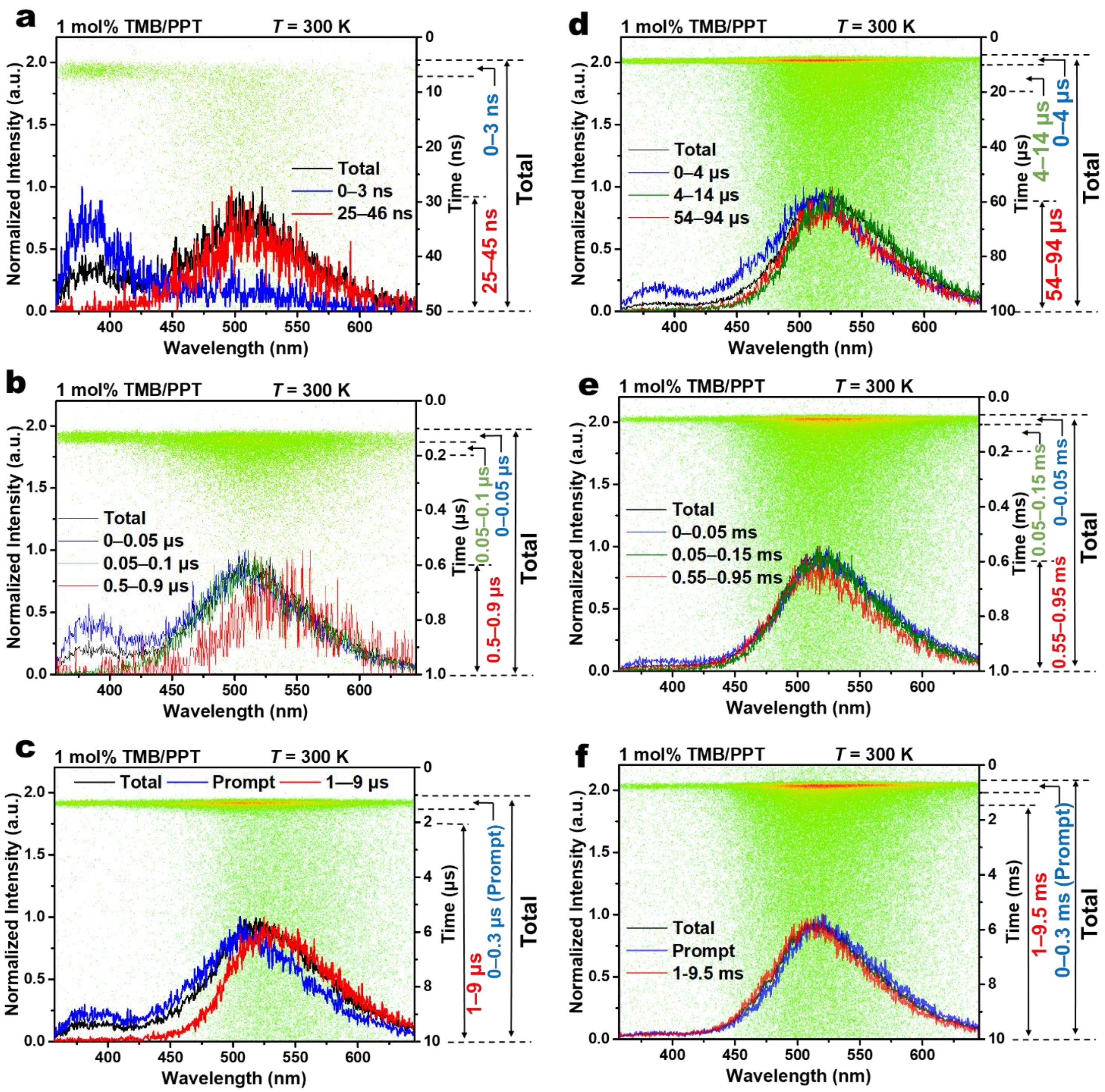

Supplementary Figure 5. Transient emission data of 1 mol% TMB/PPT film. Streak images and transient emission spectra on different time scales at 300 K in vacuum. **a.** 50 ns; **b.** 1 μs; **c.** 10 μs; **d.** 100 μs; **e.** 1 ms; **f.** 10 ms.

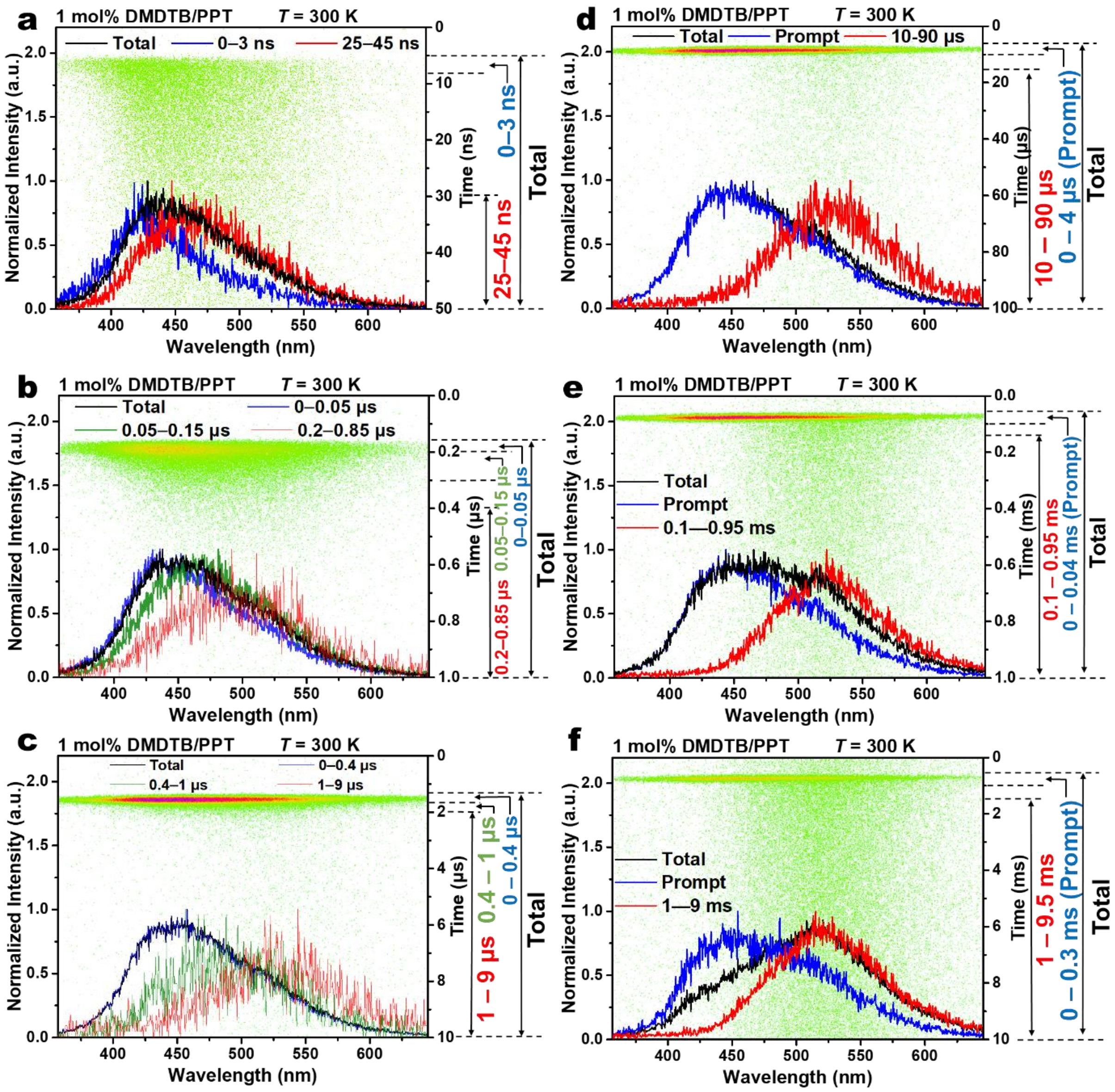

Supplementary Figure 6. Transient emission data of 1 mol% DMDTB/PPT film. Streak images and transient emission spectra on different time scales at 300 K in vacuum. **a.** 50 ns; **b.** 1 μs; **c.** 10 μs; **d.** 100 μs; **e.** 1 ms; **f.** 10 ms.

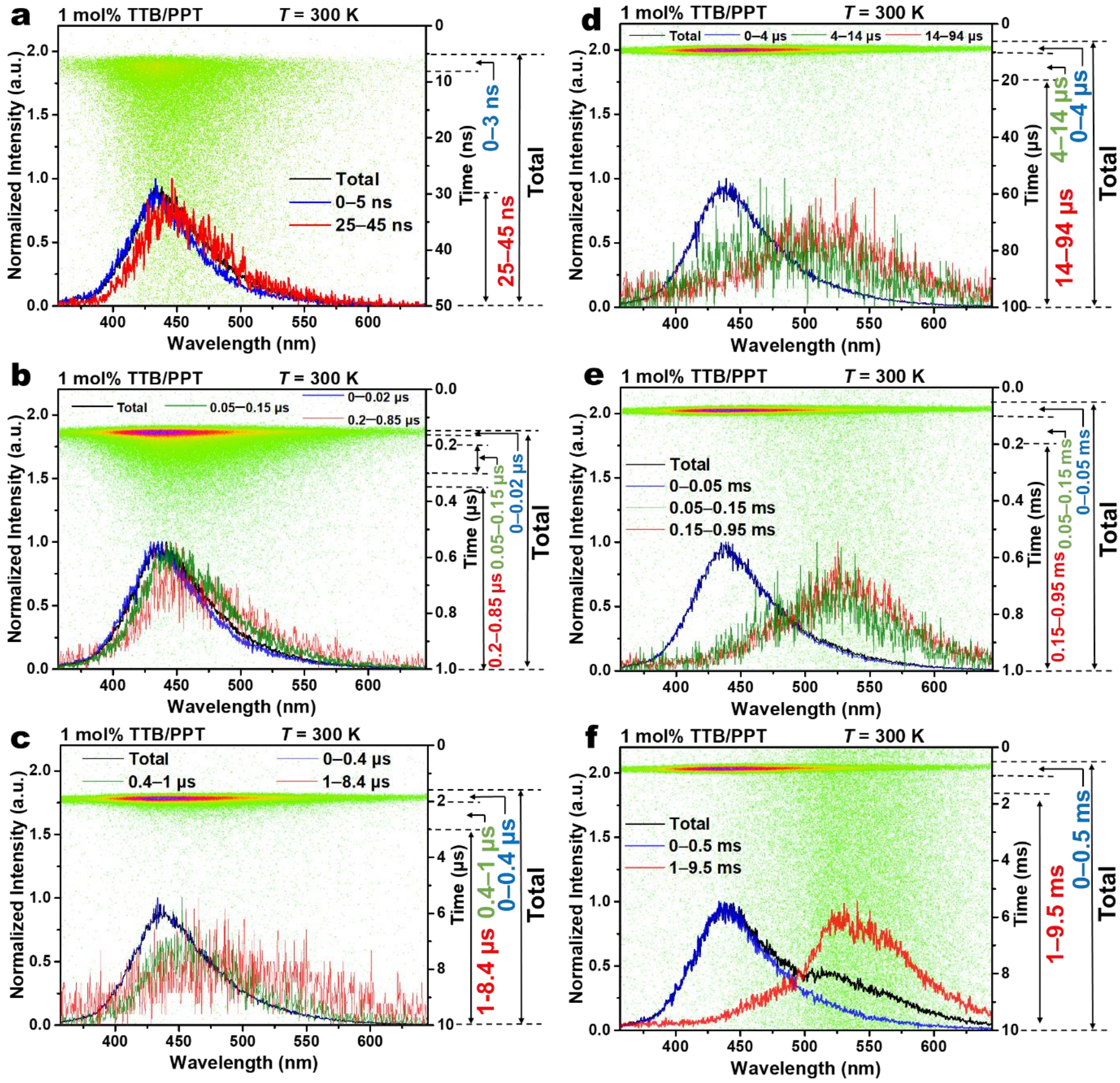

Supplementary Figure 7. Transient emission data of 1 mol% TTB/PPT film. Streak images and transient emission spectra on different time scales at 300 K in vacuum. **a.** 50 ns; **b.** 1 μs; **c.** 10 μs; **d.** 100 μs; **e.** 1 ms; **f.** 10 ms.

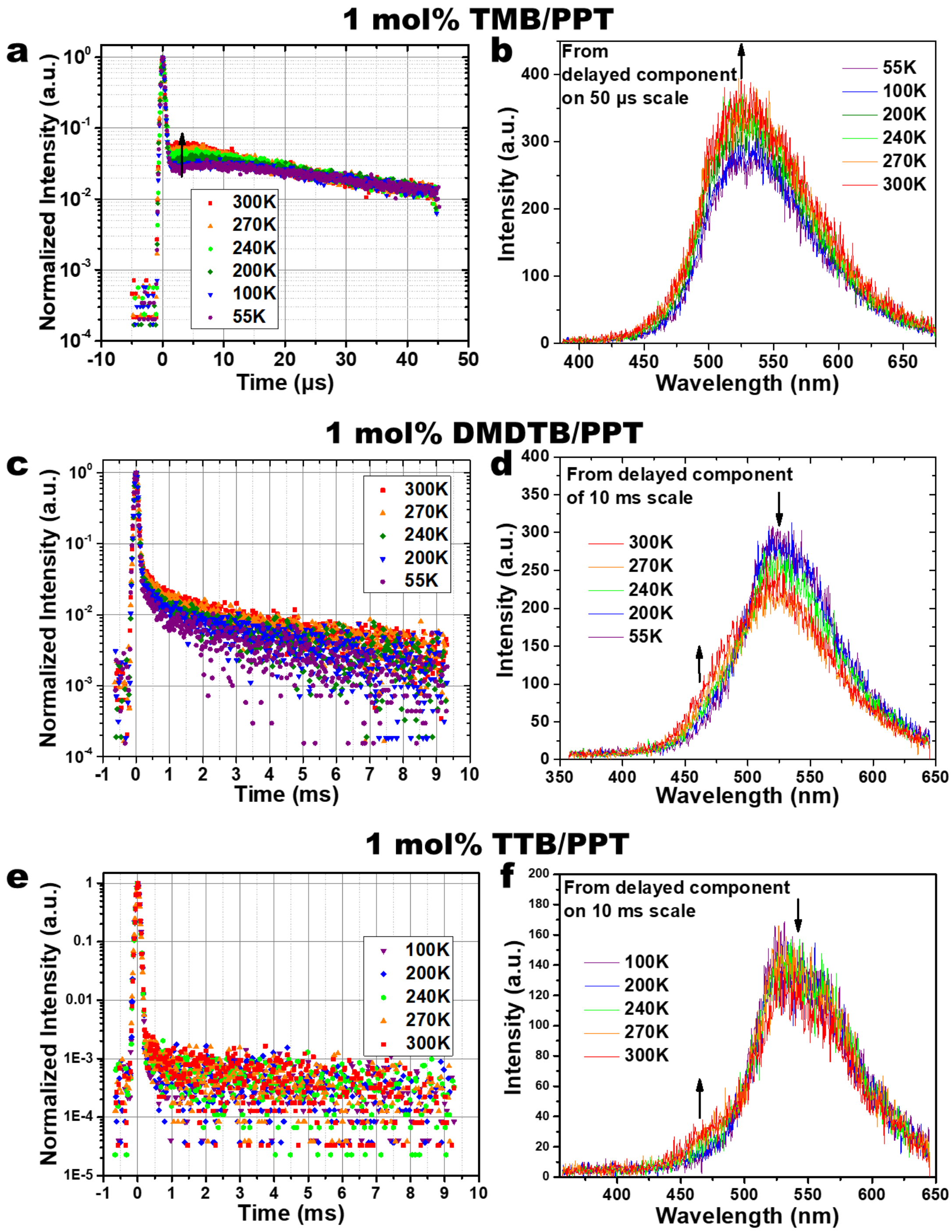

Supplementary Figure 8. Temperature-dependent emission decay profiles and emission spectra. Temperature dependence of the transient emission decay curves (semi-logarithmic plots) and emission spectra of TMB/PPT (**a, b**), DMDTB/PPT (**c, d**), and TTB/PPT (**e, f**). The emission decay profiles obtained with a streak camera system (Supplementary Figures. 5–7) were integrated over the emission from 400 to 650 nm. All graphs normalized by prompt emission peak intensity measured for the respective time range. Because differences in resolution on different time scales lead to different prompt emission peaks, comparison among different time ranges based on the absolute values is difficult.

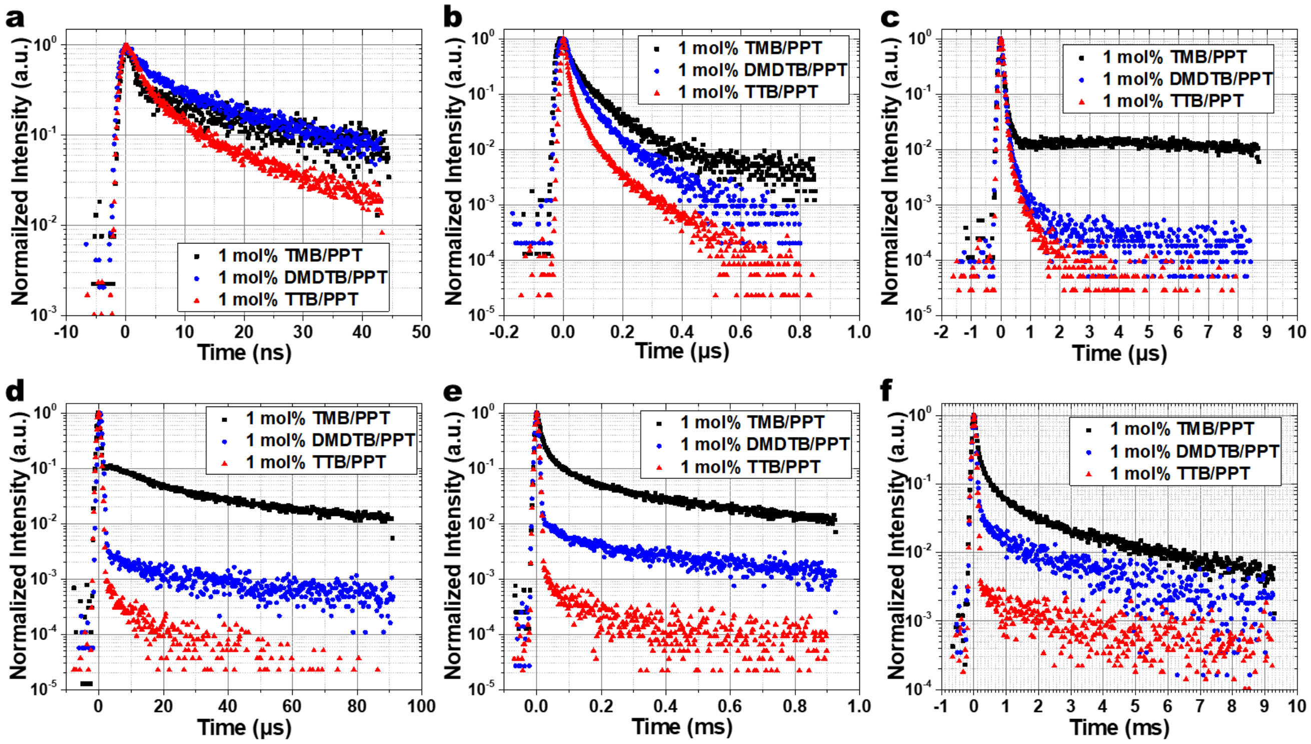

Supplementary Figure 9. Transient emission decay profiles on different time scales. Transient emission decay curves (semi-logarithmic plots) of TMB/PPT, DMDTB/PPT, and TTB/PPT on different time scales at 300 K. **a.** 50 ns; **b.** 1 µs; **c.** 10 µs; **d.** 100 µs; **e.** 1 ms; **f.** 10 ms. The emission decay profiles obtained with a streak camera system (Supplementary Figures. 5–7) were integrated over the emission from 400 to 650 nm. All graphs normalized by prompt emission peak intensity measured for the respective time range. Because differences in resolution on different time scales lead to different prompt emission peaks, comparison among different time ranges based on the absolute values is difficult.

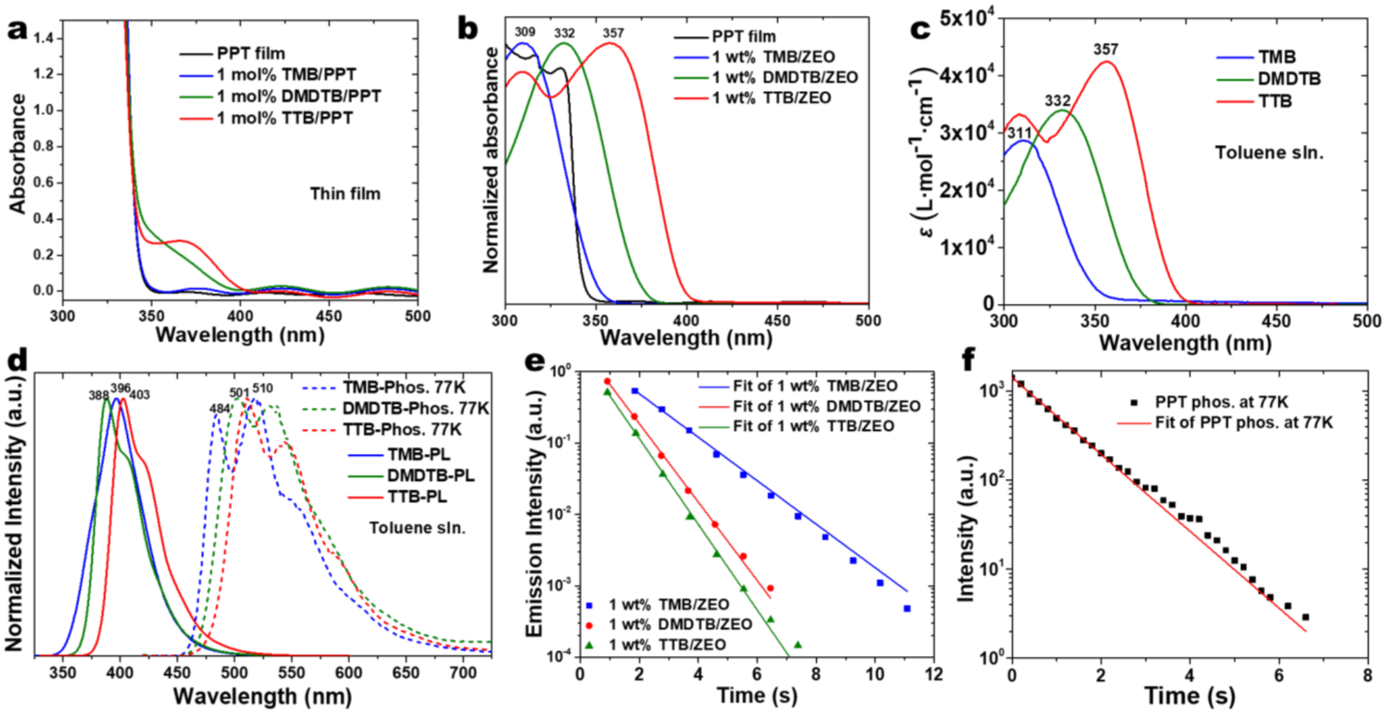

Supplementary Figure 10. Photophysical properties of donor materials. **a.** UV–vis absorption spectra of PPT, TMB/PPT, DMDTB/PPT, and TTB/PPT. **b.** UV–vis absorption spectra of PPT, TMB/ZEONOR, DMDTB/ZEONOR, and TTB/ZEONOR. **c.** UV–vis absorption spectra of TMB, DMDTB, and TTB in the dilute toluene solutions (10^{-5} M). ε is the molar extinction coefficient. **d.** Steady-state photoluminescence spectra at 300 K and phosphorescence at 77 K of TMB, DMDTB, and TTB in toluene solutions (10^{-5} M). **e.** Phosphorescence decay profiles of TMB/ZEONOR, DMDTB/ZEONOR, and TTB/ZEONOR at 300 K. **f.** Phosphorescence decay profiles of a PPT thin film at 77 K.

Supplementary Table 2. Calculated dipole moments of PPT, TMB, DMDTB, and TTB in S_0, S_1, T_1, and radical states.

Dipole moment (Debye)	S_0	S_1	T_1	Radical ion
PPT	4.82	3.50	3.45	26.89 [a)]
TMB	0.16	0.0084	0.0018	21.22 [b)]
DMDTB (conformer A) [c)]	0.12	0.93	0.17	9.74 [b)]
DMDTB (conformer F) [c)]	2.57	0.51	1.30	18.08 [b)]
TTB	0.0084	0.036	0.012	13.06 [b)]

[a)] Radical anion; [b)] Radical cation. [c)] Calculations based on conformers at the ground state as shown in Supplementary Figure 15. Level of theory: DFT-PBE0, basis set: ma-Def2-TZVP. The excited state of the exciplex always exhibits a large dipole moment.[2]

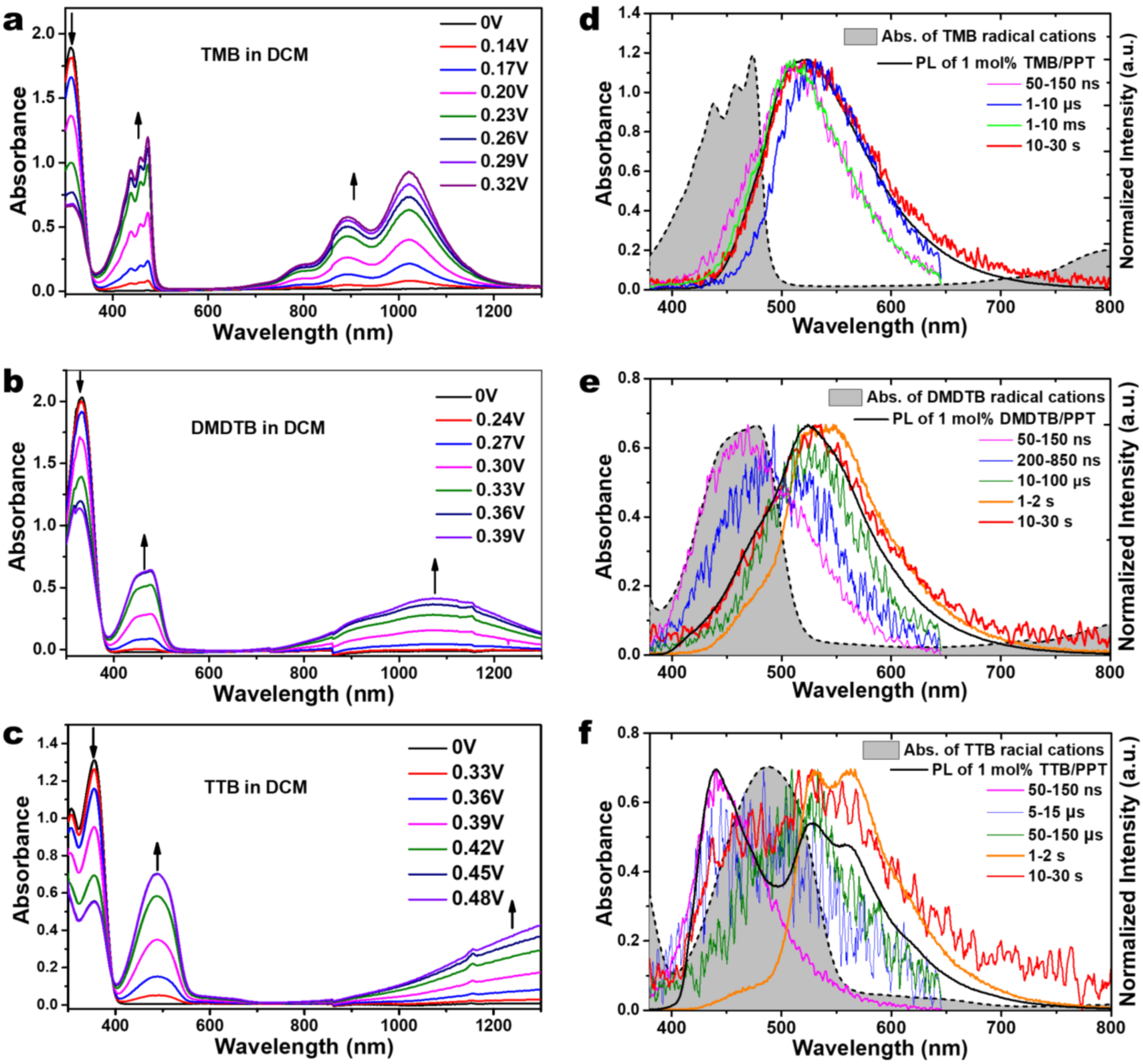

Supplementary Figure 11. Absorption spectra of donors under oxidation. **a–c.** Changes in absorption spectra for TMB, DMDTB and TTB during the first oxidation at various applied potentials in CH_2Cl_2 containing 0.1 M $TBAPF_6$. **d–f.** UV–vis absorption spectra of radical cations of the three donors [obtained from **a–c**] and the steady-state photoluminescence, LPL, and some transient luminescence spectra of three doped PPT films at 300K.

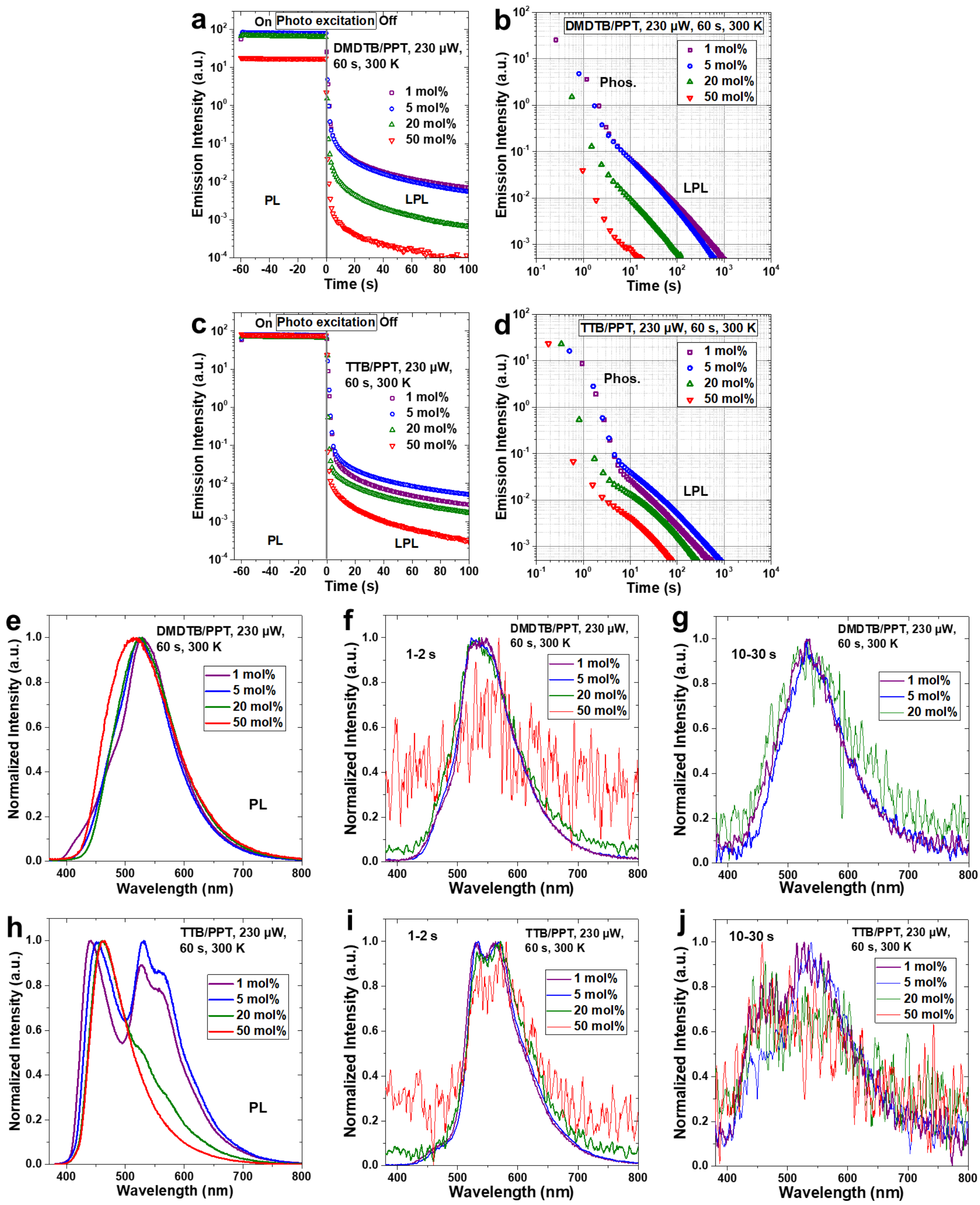

Supplementary Figure 12. Concentration-dependence of emission properties. Semi-logarithmic plots (**a, c**) and logarithmic plots (**b, d**) of the emission decay profiles, steady-state photoluminescence spectra (**c, h**) and time-resolved spectra (**f, g, i, j**) of DMTDB/PPT, and TTB/PPT with different donor concentration. Samples were excited for 60 s by a 340-nm LED source with the same power 230 μW at 300 K. PL means the steady-state photoluminescence, LPL means the long-persistent luminescence, Phos. means the phosphorescence. The time-resolved spectra were integrated over periods of 1–2 and 10–30 s after stopping excitation.

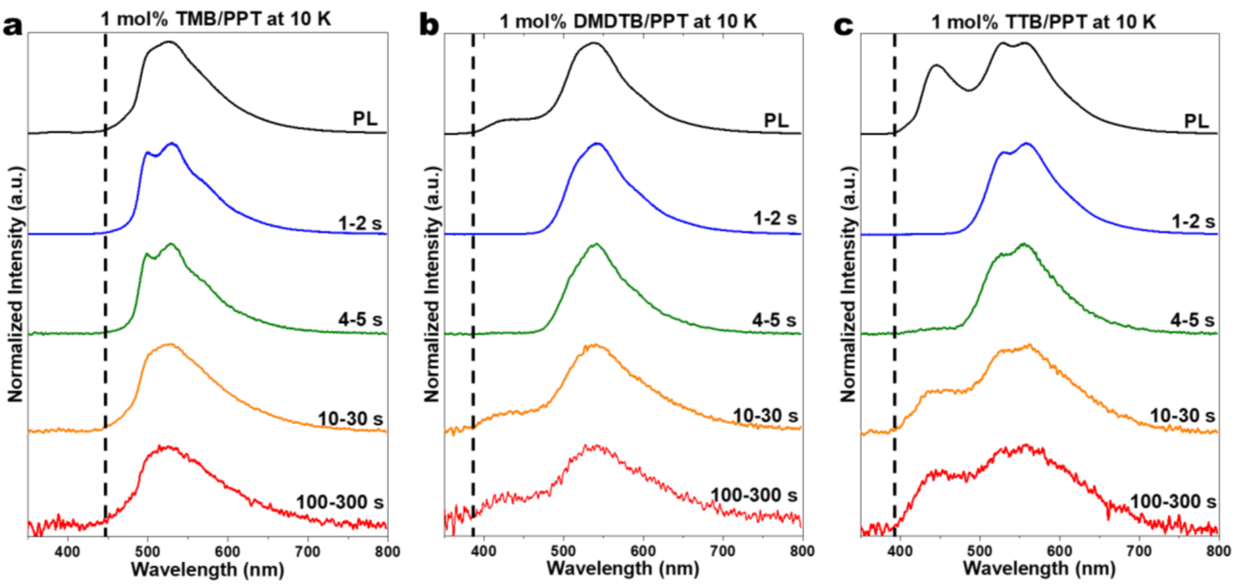

Supplementary Figure 13. Time-resolved emission spectra at 10 K. The steady-state photoluminescence (PL) and time-resolved photoluminescence spectra of 1 mol% TMB/PPT (**a**), DMDTB/PPT (**b**), and TTB/PPT (**c**) films at 10 K. The time-resolved spectra were integrated during the periods of 1–2, 4–5, 10–30, and 100–300 s after stopping the excitation. The dashed lines indicate the onset of the spectra.

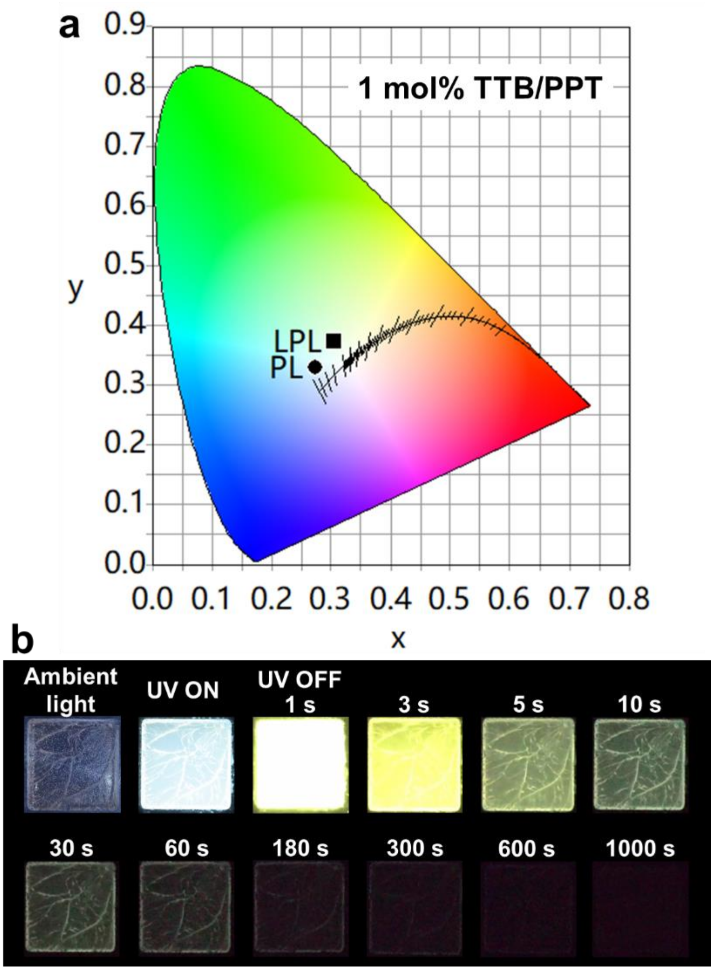

Supplementary Figure 14. Emission color of TTB/PPT. **a.** CIE 1931 coordinates of photoluminescence (0.27, 0.33) and LPL (0.31, 0.37) of 1 mol% TTB/PPT. **b.** Photograph of 1 mol% TTB/PPT film at 300 K.

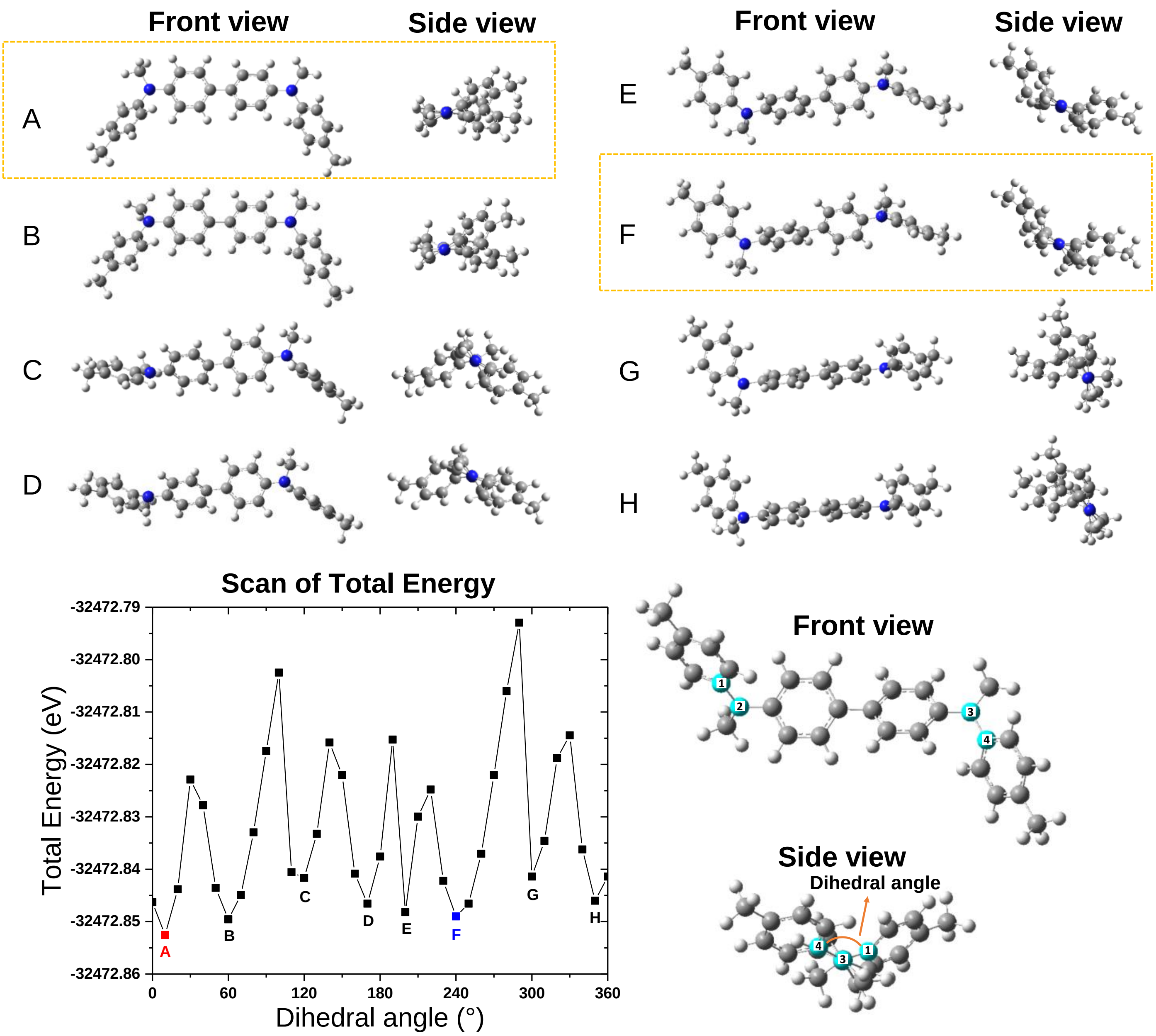

Supplementary Figure 15. Calculated molecule-conformation of DMDTB. The potential energy surface and the conformations of DMDTB at the ground state in vacuum at the B3LPY/6-31G level (refer to the method of Supplementary Reference 3). The dipole moments of conformers A and F were calculated using DFT at the PBE1PBE/ma-Def2-TZVP level.

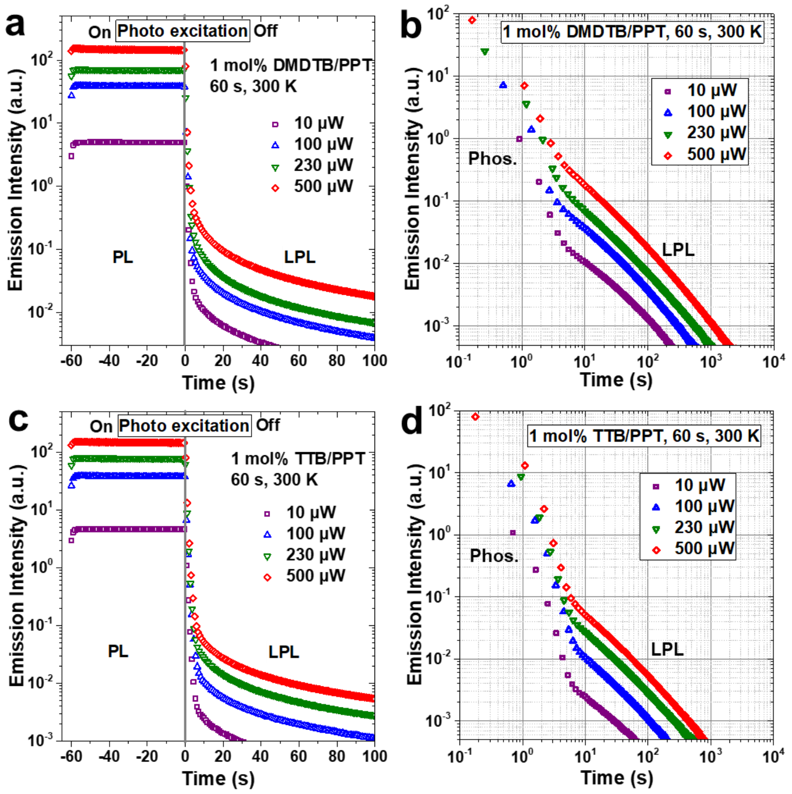

Supplementary Figure 16. Excitation power dependence of emission properties. Semi-logarithmic plots (**a, c**) and logarithmic plots (**b, d**) of the emission decay profiles of 1 mol% DMTDB/PPT and 1 mol% TTB/PPT with different excitation powers. Samples were all excited for 60 s (from −60 to 0 s) by a 340-nm LED source at 300 K. PL means the steady-state photoluminescence, LPL means the long-persistent luminescence, and Phos. means the phosphorescence.

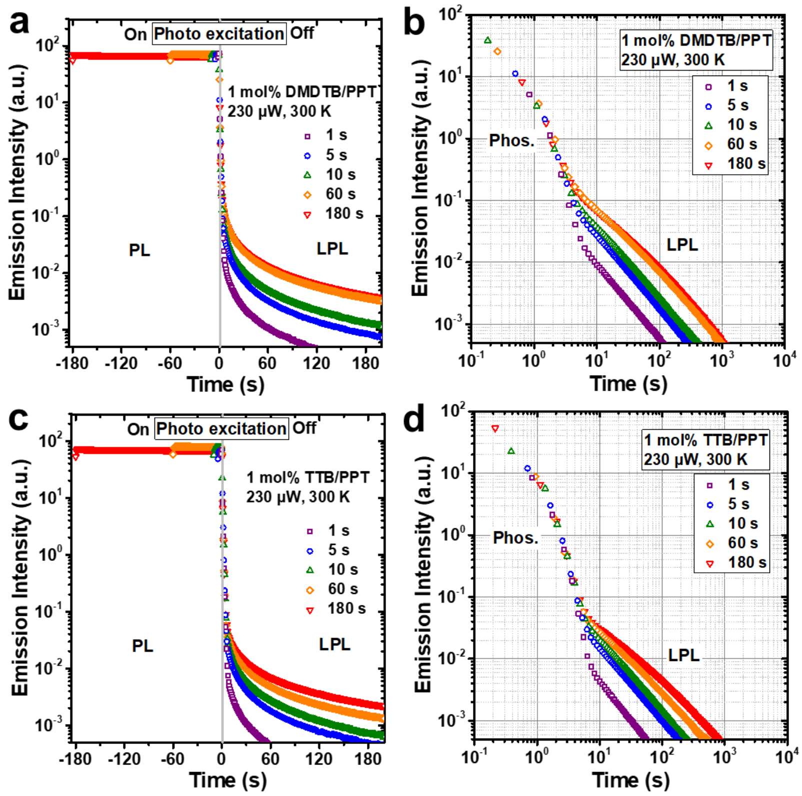

Supplementary Figure 17. Excitation time dependence of emission properties. Semi-logarithmic plots (**a, c**) and logarithmic plots (**b, d**) of the emission decay profiles of 1 mol% DMTDB/PPT and 1 mol% TTB/PPT with different excitation times. Samples were all excited by a 340-nm LED source with a power 230 μW at 300 K. PL means the steady-state photoluminescence, LPL means the long-persistent luminescence, and Phos. means the phosphorescence.

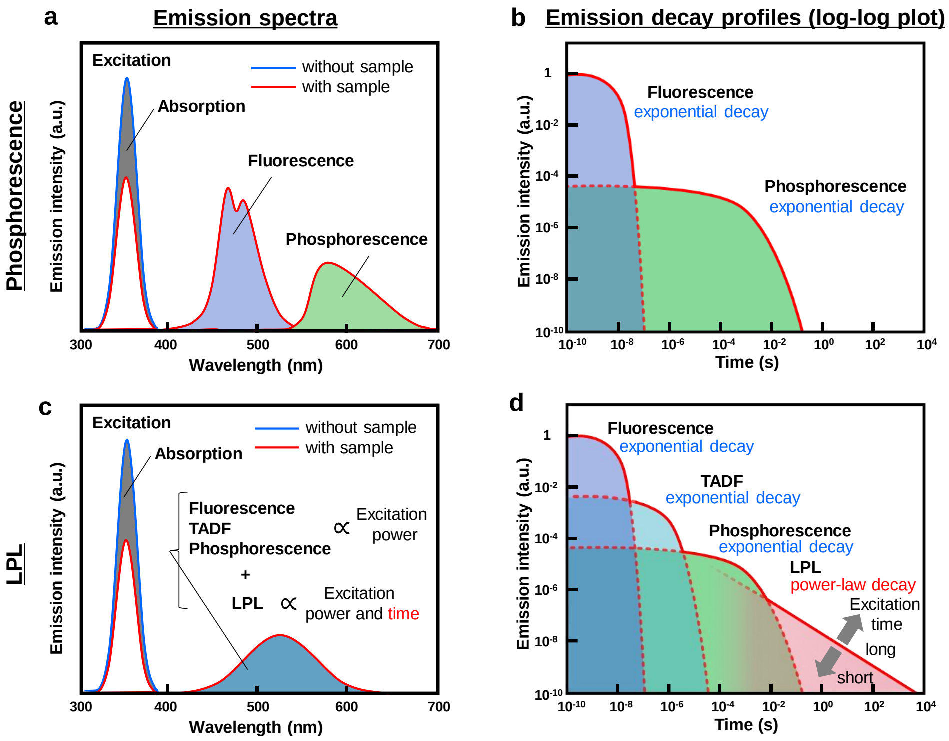

Supplementary Figure 18. Differences between phosphorescence and LPL. Ideal emission spectra and logarithmic plots of the emission decay profiles of phosphorescent materials (**a, b**) and OLPL materials (**c, d**). The phosphorescence quantum yield can be calculated from the areas under the absorption and phosphorescence emission spectra (**a**). If the fluorescence and phosphorescence spectra have a large overlap, phosphorescence quantum yield can be estimated from the emission decay profiles because both fluorescence and phosphorescence follow an exponential decay (**b**). The OLPL system exhibits fluorescence, TADF, phosphorescence, and LPL from similar energy levels (**c**). Although the fluorescence, TADF, and phosphorescence follow an exponential decay, the power-law decay of LPL makes it difficult to estimate the LPL contribution since it depends on the excitation time as well as power (**d**).

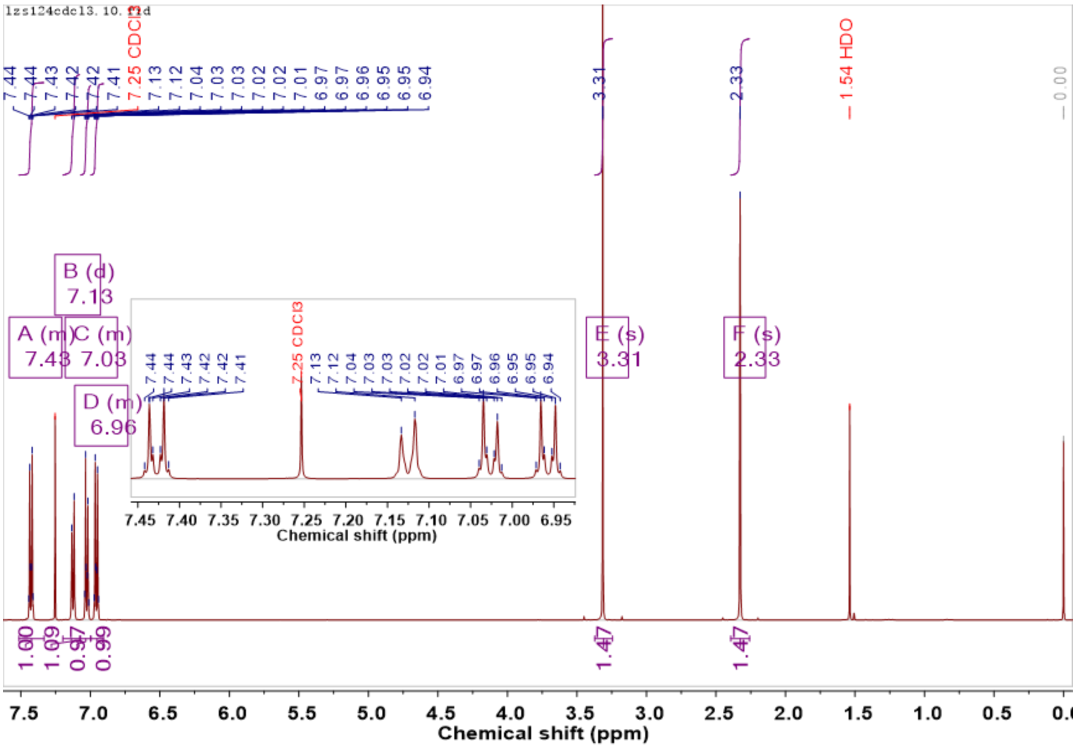

Supplementary Figure 19. ^{1}H NMR spectra of DMDTB.

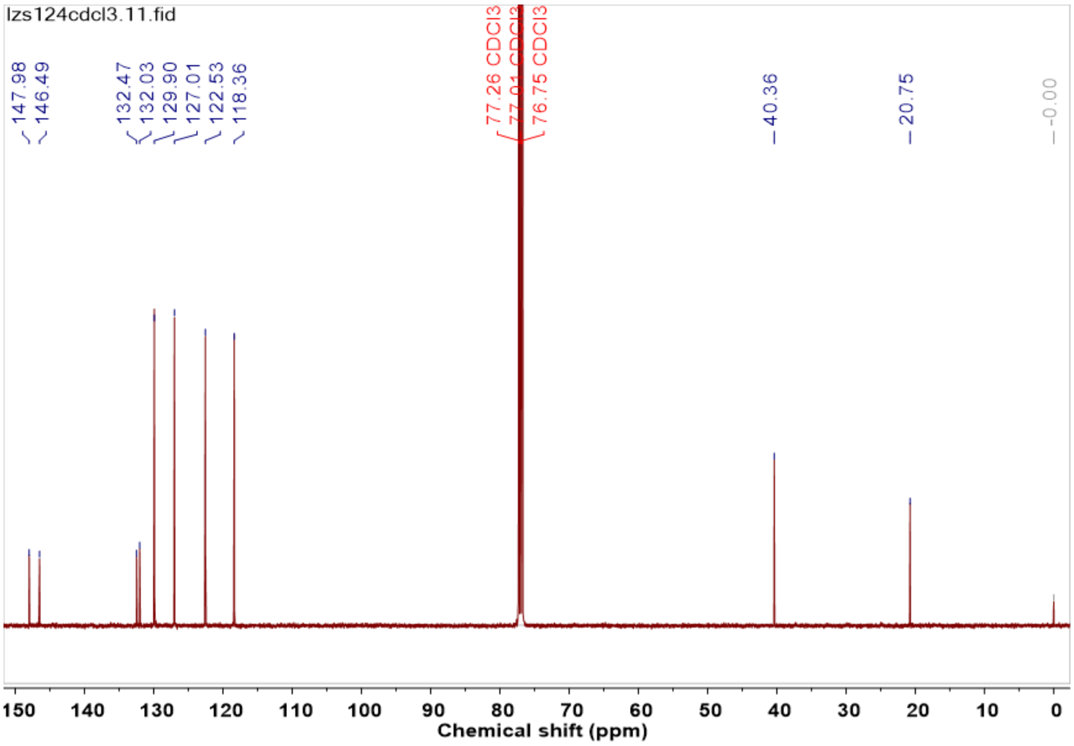

Supplementary Figure 20. ^{13}C NMR spectra of DMDTB.

Supplementary References

1 Jinnai, K., Nishimura, N., Kabe, R. & Adachi, C. Fabrication-method independence of organic long-persistent luminescence performance. *Chem. Lett.* **48**, 270-273 (2019).

2 Mylon, S. E., Smirnov, S. N. & Braun, C. L. Exciplex Dipole Moments: Cyanoanthracene Acceptors and Methyl-Substituted Benzene Donors. *J. Phys. Chem. A* **102**, 6558-6564 (1998).

3 Wang, K. *et al.* Avoiding Energy Loss on TADF Emitters: Controlling the Dual Conformations of D–A Structure Molecules Based on the Pseudoplanar Segments. *Adv. Mater.* **29**, 1701476 (2017).
