# Supplementary material for: Influence of energy gap between charge-transfer and locally excited states on organic long persistence luminescence
Source: Nat Commun. 2020 Jan 10;11:191. doi: 10.1038/s41467-019-14035-y (PMC6954229; doi:10.1038/s41467-019-14035-y)
Supplement: Supplementary file 3 — Description of Additional Supplementary Files [file 41467_2019_14035_MOESM3_ESM.docx]

**Description of Supplementary Files**

File Name: Supplementary Information

Description: Supplementary Methods, Supplementary Figures, Supplementary Tables and Supplementary References

File Name: Supplementary Movie 1
Description: The appearance of 1 mol% TTB/PPT film excited by 365 nm UV lamp for 5 min at 300 K.
